# Heritability estimates of distichiasis in Staffordshire bull terriers using pedigrees and genome-wide SNP data

**DOI:** 10.1186/s13028-022-00650-1

**Published:** 2022-11-21

**Authors:** Dina Joergensen, Per Madsen, Ernst-Otto Ropstad, Frode Lingaas

**Affiliations:** 1 Department of Preclinical Sciences and Pathology, Faculty of Veterinary Medicine, Norwegian University of Life Sciences, NMBU Veterinærhøgskolen, Oluf Thesens Vei 22, 1433 Ås, Norway; 2 Center for Quantitative Genetics and Genomics, Aarhus University, 8830 Tjele, Denmark; 3 Evidensia, Oslo Dyresykehus, Ensjøveien 14, 0655 Oslo, Norway

**Keywords:** Aberrant eyelash, Canine, Genomic heritability, Ocular disorder, SNP-based heritability

## Abstract

**Background:**

Distichiasis is the most frequently recorded eye disorder in the Norwegian Staffordshire bull terrier (SBT). The condition is often mild but can, in severe cases, lead to pain and blindness. The current study's main purpose was to estimate the heritability based on pedigree information as well as single nucleotide polymorphisms (SNPs) to evaluate whether it is realistic to reduce the frequency by systematic breeding. The majority of the dogs had only one examination as a young puppy. To evaluate whether this early screening gave a reliable representation of the disease burden in the population, we compared the diagnosis in puppies and adult dogs.

**Results:**

Our material consisted of data from 4177 dogs with an overall prevalence of distichiasis of 8.38% (CI 7.56–9.26). The prevalence in puppies examined around eight weeks of age was significantly lower than in dogs examined after 52 weeks (2.87%, CI 2.29–3.54 versus 18.72%, CI 16.71–20.87). The heritability was estimated in dogs examined after 52 weeks. We used both pedigree (1391 dogs) and genotype (498 dogs) information for the estimates. The pedigree-based heritability was ~ 0.22 (on the underlying scale ~ 0.48), while the genomic-based heritability (on the underlying scale) was ~ 0.47, and ~ 0.37 when excluding close relatives with equal affection status.

**Conclusions:**

Screening for distichiasis in puppies before eight weeks of age is not sufficient to give an accurate estimate of the prevalence, and an additional examination after one year is recommended. The heritability of distichiasis is medium to high, showing that it should be possible to reduce the prevalence by selective breeding.

## Background

Distichiasis is a condition of the eyelid with displaced eyelashes [1]. The aberrant hairs arise from ectopic hair follicles close to the meibomian glands in the eyelid and mostly emerge from the duct openings of the meibomian glands on the margin of the eyelid as single or multiple hairs [2, 3]. The disorder is common in dogs but has also been reported in cats [4], ferrets [5], and horses [6]. It is seen in both purebred and mixed-breed dogs [1, 2, 7]. The prevalence varies strongly between breeds, with 49.3% in English cocker spaniels [8], 11.4% in Tibetan terriers [9], and 27.9% in Elos [10]. Distichiasis is the most frequently diagnosed ocular disorder in the Norwegian Staffordshire bull terrier (SBT) population [11].

Distichiasis occurs in puppies as young as six weeks of age [12]. The majority of dogs diagnosed with distichiasis are only mildly affected, with subtle or no apparent clinical signs except for aberrant eye hair. Common clinical signs are conjunctivitis, irritation and rubbing of the eye, increased blinking, and lacrimation. In severe cases, the abnormal hair growth can lead to corneal lesions such as ulceration and keratitis [1, 12, 13]. There are several different treatment methods, but recurrence and complications are associated with all procedures [3, 13–15]. Therefore, a reduction of the incidence by selective breeding would be advantageous.

Heritabilities (*h*^*2*^) for distichiasis have been reported for: the Tibetan terrier (*h*^*2*^ = 0.043, 849 dogs) [9], Elo (*h*^*2*^ = 0.238 ± 0.122, 234 dogs) [10], English cocker spaniels (*h*^*2*^ = 0.22 and 0.51, 799 dogs) [8], and Havanaise (*h*^*2*^ = 0.276 linear model and *h*^*2*^ = 0.720 Bayesian threshold model, 1156 dogs) [16].

The main purpose of the present study was to estimate the additive heritability (*h*^*2*^) and the prevalence of distichiasis in the SBTs in Norway and to explore whether it is realistic to reduce the incidence of distichiasis by selective breeding. In addition, we were interested in investigating the possibility of using genomic data to estimate the heritability of distichiasis in dogs. So far, the heritability of distichiasis has been estimated by using pedigree information only, and to the best of our knowledge, this is the first study to include genomic data in heritability estimates of an ocular disorder in dogs.

## Methods

The study was based on official data from eye examination records registered by the Norwegian Kennel Club (NKK). ECVO-certified veterinarians performed the examinations using a bio-microscopical examination of the adnexal structures of the eye. The results were stored and are publicly available in "Dogweb",—a pedigree database maintained by the NKK (www.dogweb.no).

The primary dataset contained records from 2005 until May 2021 and comprised a total of 4752 eye examinations recorded in 4177 SBTs. 499 (10.5%) dogs had more than one eye examination (the cumulative numbers were: 1 dog = 5 examinations, 11 dogs = 4 examinations, 64 dogs = 3 examinations, and 499 dogs = 2 examinations). All dogs, both uni- and bilaterally affected, were counted once. The proportion of female dogs examined was 2196 (53%) and male dogs 1981 (47%). The age of the examined dogs ranged from 4.5 weeks to twelve years. 2894 (69%) of the dogs were examined before 58 days (~ 8 weeks).

At present, a positive distichiasis diagnosis recorded in the NKK will persist, regardless of the findings on later examinations. The affected dogs are graded as mildly or severely affected; earlier, the grade moderately affected was also included. We have treated the phenotype as a binary trait (affected/unaffected) due to missing categorisation in about 16% of the affected dogs, low numbers of the more severely affected dogs and previous practice in heritability estimates of distichiasis [8, 9, 16].

### Descriptive statistics

The descriptive statistics calculating the prevalence and the effect of age and sex were performed using base R, the R package tidyverse, and epiR [17–19]. P values below 0.05 were considered statistically significant with a 95% confidence interval (CI).

The data was unbalanced, with a large group of dogs examined around eight weeks of age and several dogs with multiple examinations. Therefore, the data were stratified into three age groups: D1: dogs with a single standing examination between 0 and 58 days, D2: dogs examined between 59 and 364 days, and D3: dogs examined after 365 days (Table 1). There was missing information on the age at the examination in twelve dogs (one affected), and these were excluded from further analysis. Other age groups were assessed by splitting the dogs into six different age classes, 0–1, 1–2, 2–3, 3–4, 4–5 and > 5 years. The oldest age groups (dogs > 5 years) were merged due to small numbers of observations in this age span. There was no significant difference in distichiasis status between any age groups above one year, and they were joined into one class, D3.

**Table 1:** Age distribution and the prevalence of distichiasis in the different age groups

Age group		Age (weeks)	Number of dogs	Number of dogs affected with distichiasis	Prevalence of distichiasis in %	Standard error	Confidence interval (95%)	Odds
	All Young examined ≤ 0.16 year	≤ 8.3	2894	83	2.87	0.003	2.29–3.54	0.03
D1	Young only examined once ≤ 0.16 year	≤ 8.3	2508	76	3.03	0.003	2.39–3.78	0.03
D2	Last examination between > 0.16 < 1 year	8.3–52	263	12	4.56	0.013	2.38 -7.83	0.05
D3	All dogs examined after one year	≥ 52	1394	261	18.72	0.01	16.71—20.87	0.23

The distribution of the examined dogs in the different age groups: All dogs < 0.16 years, D1, D2 and D3. The prevalence of distichiasis is given in the four age groups, including the standard error and the 95% confidence interval of the prevalence. The last column shows the odds of being affected with distichiasis in the different age groups

The effect of age and sex between the two groups D1 and D3 were estimated using logistic regression, including distichiasis as a response variable and the age groups and sex as the explanatory variables. We also compared the diagnosis in dogs with multiple examinations, once before 58 days and at least one examination after 364 days using the McNemar's test.

The effect of age at examination within the age groups D2 and D3 was estimated using logistic regression, including distichiasis as a response variable and age as the explanatory variable. Only one observation per dog was used. In dogs with multiple examinations, we used the age at the last presentation in unaffected and the age at first positive diagnosis in affected dogs. In case of inconsistency in the distichiasis status between two examinations, the more severe diagnosis was kept, affected individuals were considered once affected, always affected.

### Estimates of pedigree-based heritability

The pedigree-based heritability (*h*^*2*^_*ped*_) estimating additive effects was conducted on 1391 dogs in age group D3. The youngest (D1) and the middle age group (D2) were excluded from further analysis due to the low prevalence in D1 and the low number of observations in D2.

The *h*^*2*^_*ped*_ was estimated in the age group D3 using an average information restricted maximum likelihood approach (REML), analysed in the DMU package [20]. Afterwards, the heritability was converted to a theoretical underlying continuous scale (*h*^*2*^_*pedT*_) [21]. The model used was Y = μ + a + e. Where Y = distichiasis status, μ = the mean term (fixed effect), a = the additive genetic effect (random effect), and e = the residual error (random effect).

### Genomic heritability

The biological material was based on samples from a biobank established in collaboration between The Norwegian University of Life Sciences and the NKK. DNA was extracted using E.Z.N.A Blood DNA Mini Kit from Omega. The quality of the DNA was measured using Epoch from BioTek. A total of 681 dogs were genotyped (118 dogs with Illumina 170k CanineHD Bead chip and 629 dogs with Illumina 220k CanineHD Bead chip). All material was gathered in agreement with all relevant ethical guidelines and with the owners' written consent.

### Quality control

Plink 1.9 and R were used for data management and quality control (QC) [17, 22, 23]. We performed a QC on each dataset before merging. Dogs with more than 5% missingness, a heterozygosity rate above three standard deviations from the mean, sex mismatches and duplicates were removed. Markers with a call rate below 95% and a minor allele frequency of ≤ 0.04 were removed. After QC, 611 dogs and 129,217 markers from the 220k and 92 dogs and 101,806 markers remained in the 170k dataset. After merging the two datasets, only markers in common between the two datasets were kept (93,973 markers), and a post-merge QC was performed with the same parameters as pre-merge QC, removing 37 markers with a minor allele frequency below 0.04 and 20 duplicated individuals. Multidimensional scaling plots were conducted to inspect potential differences (batch effects) between the two datasets (170k and 220k). Only dogs in D3 were kept for further analyses to make the genomic heritability estimates comparable to the pedigree-based estimates, excluding 133 individuals. The final dataset consisted of 93,936 markers and 548 dogs, where 228 were affected, and 320 were unaffected.

### Genomic heritability estimates

The genomic heritability (*h*^*2*^_*g*_) was estimated using a genomic restricted maximum likelihood (GREML) model in Genome-wide Complex Trait Analysis software (GCTA). Using the model: Y = μ + g + e, where Y = distichiasis status, μ = the mean term, g = the genetic effect based on the genomic relationship matrixes (GRM), and e = the residual. The variance estimates explained on the observed scale are transformed by GCTA to a modified version of the underlying scale adjusting for sample ascertainment caused by an increased number of cases in the sample compared to the actual population [21, 24].

As there is little knowledge about the genetic architecture of distichiasis, two GRMs were used. The first was calculated in GCTA, where the GRM is calculated from autosomal single nucleotide polymorphisms (SNP), and the SNPs are assumed to contribute an equal amount to the trait [24]. Due to the high level of linkage disequilibrium (LD) in dogs [25], we calculated an alternative GRM in Linkage Disequilibrium Adjusted Kinship software (LDAK). In LDAK, SNPs are weighted depending on the degree of LD in the region. SNPs in regions with high levels of LD receive a lower weight than SNPs in regions with a lower LD level, thus avoiding underestimating causal variants in areas with low levels of LD and overestimating variants in areas with high levels of LD [26].

To look at potential biases introduced by closely related individuals, we ran one analysis including all individuals and one analysis where siblings with equal affection status were removed (50 dogs). In sibling pairs with both affected and unaffected siblings, one of each was kept (36 sibling pairs), leaving 207 affected and 291 unaffected individuals. The disease prevalence was set to 0.187 according to the prevalence among dogs examined after one year of age.

## Results

### Prevalence and grading

**Fig. 1:**
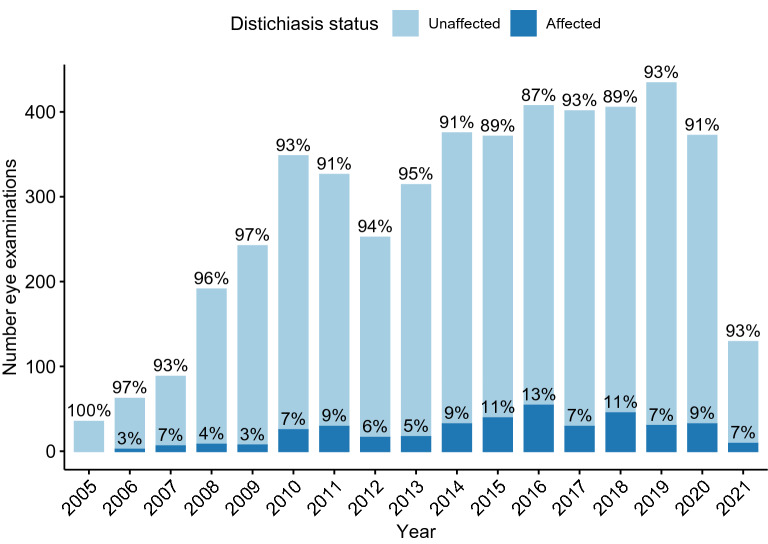
Number of eye examinations pr year

The proportion of dogs diagnosed with distichiasis each year ranged from 2.89% (2009) to 13.5% (2016) (Fig. 1). The total number of dogs diagnosed with distichiasis was 350, giving an overall prevalence of 8.38% (CI 7.56–9.26). The majority, 256 (73.14%) of the affected dogs, were marked as mildly affected, 31 (8.86%) dogs as moderate and, seven (2%) dogs severely affected, 56 (16%) dogs had no grading marked on the examination scheme.

### Effect of age and distribution

**Fig. 2:**
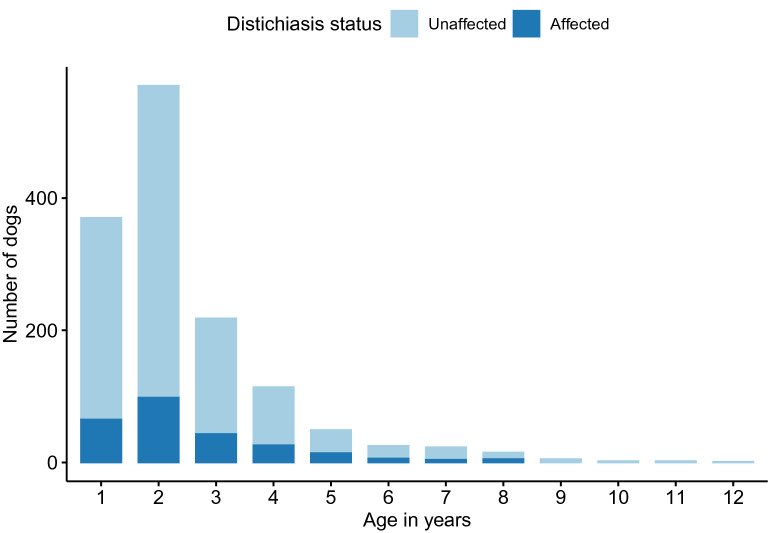
Age distribution of dogs examined after 1 year of age

The age distribution is displayed in Table 1 and Fig. 2. The prevalence of distichiasis was significantly higher in D3 (18.72%) compared with D1 (3.03%). Thus, there is a significantly increased risk of being diagnosed with distichiasis in D3 compared to D1 (P =  < 2e−16, CI 1.71–2.24). Comparing 370 dogs examined both before 53 days and after one year of age gave a significantly increased risk of being diagnosed with distichiasis at the second examination, after one year of age (62 affected dogs) compared with the first examination (6 affected dogs) (P = 9.41e−15). There was a significantly increased risk of distichiasis with increasing age within D2 (P = 3.89e−05, CI 2.17–5.98), but not in D3 (P = 0.9, CI − 0.10 to 0.08).

### Re-examination

The diagnostic consistency among the 499 dogs with multiple examinations was investigated. The diagnosis changed in 82 (16.43%) of the re-examined dogs. Three dogs were first diagnosed with distichiasis, then diagnosed as free, while 79 dogs were first classified as unaffected and then as affected on a later examination. Most of these dogs (n = 63; 12.62%) were younger than eight weeks at the first examination, while only 11 dogs (2.2%) were older than one year at the initial assessment. In most cases, the grading is consistent, only two out of 350 affected dogs changed from mild to moderate, and one dog changed from moderately to mildly affected.

### Sex

**Fig. 3:**
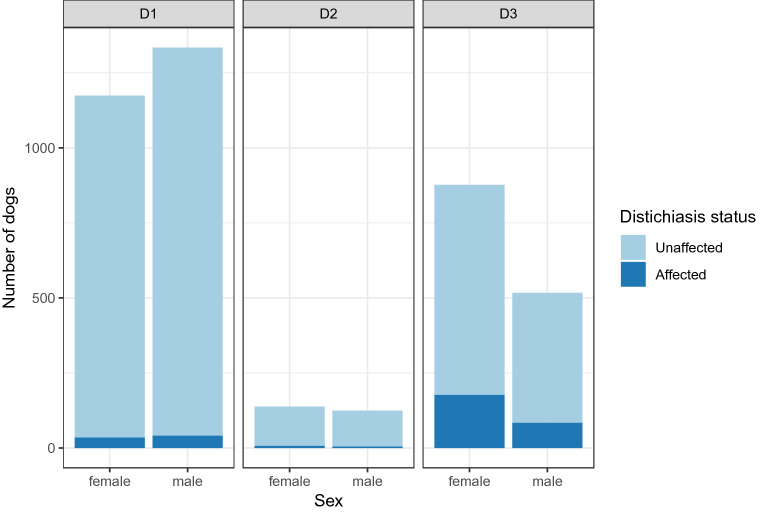
The distribution of sex in the age groups. D1 (0 < days ≤ 58), D2 (58 > days < 365) and D3 (days ≥ 365)

The number of female cases was 220, and male cases were 130 (Fig. 3). There was a greater number of female dogs (877) examined after one year of age than males (517). Female dogs were also more frequently re-examined than male dogs; 301 females had a second examination as opposed to only 198 males. The proportion of affected dogs in the two sexes was approximately the same in the three age groups. The logistic regression model showed no significant effect of sex when correcting for age (P = 0.14, CI − 0.43 to 0.06).

### Estimates of heritability

The additive heritability estimate using pedigree data (age group D3, linear model) was on the observed scale *h*^*2*^_*pedO*_ ~ 0.22, SE 0.05, and after transforming to the underlying scale, *h*^*2*^_*pedT*_ ~ 0.48, SE 0.11. The genomic heritability estimates using the different GRMs and the two different models, including all dogs and excluding siblings of equal affection status, are represented in Table 2. The average estimated genomic heritability in age group D3 was on the underlying scale *h*^*2*^_*gT*_ ~ 0.47 SE 0.10, and after removing siblings, *h*^*2*^_*gT*_ ~ 0.37 SE 0.11.

**Table 2:** The genomic heritability estimates including all models in age group D3

GRM	Model	Heritability estimates observed scale (*h*^*2*^_*gO*_)	Standard error	Heritability estimate transformed scale (*h*^*2*^_*gT*_)	Standard error
GCTA	All dogs included	0.345	0.078	0.461	0.104
LDAK	All dogs included	0.357	0.079	0.476	0.106
GCTA	Full siblings with equal affection status excluded	0.275	0.082	0.368	0.109
LDAK	Full siblings with equal affection status excluded	0.281	0.083	0.375	0.112

The heritability estimates for the different models with and without full siblings, using two different genomic relationship matrices (GRM) calculated in GCTA and LDAK. *h*^*2*^_*gO*_ is the genetic heritability estimate on the observed linear scale, and *h*^*2*^_*gT*_ is the genetic heritability estimate on the transformed underlying scale

## Discussion

The current study showed a high prevalence of distichiasis in the Norwegian SBT population. Most cases were only mildly affected. More than 50% of the dogs in this study were examined at around eight weeks of age as part of a screening for inherited eye disorders. This screening captures inherited eye diseases such as distichiasis and persistent hyperplastic *tunica vasculosa lentis*/persistent hyperplastic primary vitreous (PHTVL/PHPV). Our study indicates that this early screening has a limited predictive value for a distichiasis diagnosis in grown-up dogs, and the probability of being diagnosed with distichiasis is significantly higher after one year compared with young puppies. After one year of age, there is no clear relationship between increased age and a positive diagnosis. However, the data about dogs older than five years is sparse.

Due to the low prevalence and limited predictive value in the youngest age groups, the pedigree-based heritability estimates were based on dogs examined after 52 weeks. We based our estimates on a linear model, as Bellamy et al. showed a good agreement between heritability estimates from the Bayesian threshold model and heritability from linear models converted to the underlying scale [16]. The estimated heritability of *h*^*2*^_*pedO*_ =  ~ 0.22 and *h*^*2*^_*pedT*_ ~ 0.48 on the underlying scale agrees with other studies of dogs [8, 10], even if there is some variation between breeds. While Ketteritzsch estimated a lower heritability in Tibetan terriers [9], our estimated pedigree-based heritability is slightly lower than in the study of Bellamy et al. [16].

The genomic-based heritability was estimated to be *h*^*2*^_*gT*_ ~ 0.47, and after removing siblings with equal affection status *h*^*2*^_*gT*_ ~ 0.37 on the underlying scale. There were only minor variations between the methods. Using the GRM calculated in LDAK gave a slightly higher *h*^*2*^_*gLDAK*_ value than the *h*^*2*^_*gGCTA*_ using the GRM calculated in GCTA. The genomic heritability estimates have a relatively large standard error, and a larger sample size could reduce the standard error. The genomic-based heritability is estimated on a subset of the dogs used in the pedigree bases estimates, and the estimates are on the same level as the pedigree-based heritability when siblings with equal affection status are included. This is interesting as genomic heritability estimates tend to underestimate the heritability compared to traditional methods using pedigree data [27, 28]. A possible bias in our genomic heritability estimates is the degree of relationship in the data. In humans, genomic heritability estimates usually only include unrelated individuals [24, 27]. Within a dog breed, the average relationship is usually much higher than in humans. Removing unrelated individuals would lead to a low sample size and decreased power. Including close relatives can bias the results upwards due to a shared environment, but epistasis and dominance might also have an effect [29–31]. We removed sibling pairs with the same affected state to account for some biases introduced by close relationships. Including all siblings in the calculation increased the estimates by around 10%. In livestock, it is not uncommon to combine pedigree and genomic data in a single-step analysis to give a more accurate heritability estimate without having the cost of genotyping the whole breeding stocks [32]. The single-step method is an attractive method as long as there is no genomic selection of the trait [33]. Including pedigree information in addition to the genomic data has been shown to improve the precision of heritability estimates in dairy cattle [34].

The material for the pedigree-based estimates consisted of 1391 dogs, a relatively low proportion of the total population. There is no data about the population size, but in the last years, there have been approximately 1000 newly registered SBTs per year in Norway [35]. With an overall low number of dogs examined after one year of age, we cannot be certain that our data are representative of the whole population. We assume that most dogs intended for breeding undergo at least one eye examination as an adult. Thus, we believe our results are valid for the breeding population, the genetic basis for the next generation. We have no reason to think that breeding dogs are more prone to develop distichiasis than other family dogs. As most of the affected dogs are only mildly affected with no clinical signs, distichiasis would not commonly be a reason for an eye examination.

There are several challenges related to the diagnostics of distichiasis, which might lead to a false-negative diagnosis, including patient cooperation, the experience of the veterinarians [9], and the lifecycle of the eyelash, with shedding and regrowth. Also, single hairs not detectable at the time of examination may appear later. Gómez found that previously undetected hair could emerge by manipulating the eyelid during surgery [3]. Also, Lawson noted that it is not uncommon that more delicate hair and those just starting to appear are not observed at the initial examination [1]. At last, there is the possibility that the owner intentionally removes the *distichiae.*

The overall prevalence of distichiasis seems persistent over the years. A reduction of the frequency could be expected by following the breeding advice stated by The Norwegian Terrier Club (NTC) and ECVO: excluding severely affected dogs from breeding and only breed mildly affected dogs with unaffected dogs [36, 37]. The number of newly registered SBTs has increased 19 folds in Norway over the last 20 years [35]. The rapid growth in the popularity of the SBT might have led to high pressure on the breeding stock and less stringent selection against unfavourable traits like distichiasis, thus maintaining the high prevalence of the condition. According to the NTC's breeding strategy, distichiasis is not considered a main concern, but breeders are encouraged to exercise prudence [36]. Distichiasis usually has few clinical implications and might not be the main focus of the breeders. Among the 350 affected dogs, NKK has registered 68 affected dogs used in breeding, where most of these dogs were only mildly affected (52), and no breeding dogs were severely affected [11].

The present information about the medium to high heritability of distichiasis shows that it is possible to reduce the prevalence of the disease by selective breeding. We recommend excluding all severely affected dogs, and careful consideration must be given when breeding mildly affected dogs. In a future breeding strategy, further restrictions than those implemented by NKK and NTC at present can be evaluated after carefully considering the clinical impact of distichiasis and the presence of other important traits under selection.

## Conclusions

We found a significant increase in the disease prevalence from eight weeks to one year of age, showing that screening young puppies around eight weeks is insufficient to give an accurate "lifetime" diagnosis and might underestimate the prevalence of distichiasis in the breed. Estimates of heritability based on pedigree data and genomic SNP data indicate that the heritability of distichiasis is moderate to high. This shows that it can be possible to reduce the prevalence by weighting the trait when selecting parents for breeding. Interestingly, the genome-based heritability estimates are on the same level as the pedigree-based heritability estimates, even though the sample size is small, and the standard errors are relatively high.

## Data Availability

The eye examination records and pedigree data are available through the NKKs online database, "dogweb", at: www.dogweb.no. The genomic datasets generated and analysed during the current study are available from the corresponding author on reasonable request.
